# The Proposed Bone Post-Arterial Type R Capillaries Resolve into Venous and Fatty Acid-Handling Endothelial Cells

**DOI:** 10.64898/2026.07.27.741009

**Published:** 2026-07-28

**Authors:** Sanyam Jain, Jessy Davina Joseph, Hanyu Liu, Nainita Roy, Yang Yang, Siddharth Nishant Mundapat, Radek Machan, Antonino Glaviano, Abhishek Chandra, Rinkoo Dalan, Junyu Chen, Navin Kumar Verma, Martine Cohen-Solal, Makarand V. Risbud, Aline Bozec, Anjali P. Kusumbe

## Abstract

Endothelial specialization is increasingly recognized as a fundamental regulator of tissue homeostasis, yet the cellular diversity of the skeletal vasculature remains incompletely resolved. Here, we integrate large-scale single-cell transcriptomics, cross-tissue comparisons, and imaging to comprehensively define endothelial heterogeneity across the skeleton. Our analyses demonstrate that the proposed post- arterial “type R” endothelial population is not a distinct endothelial subtype but instead comprises canonical venous endothelial cells and fatty acid-handling endothelial state. RNA velocity supports a venous continuum, while the proposed type R markers FMO2, and AQP7 lack both endothelial and skeletal specificity. The fatty acid-handling endothelial state, characterized by Lpl and Cd36 is conserved across multiple skeletal sites and non-skeletal tissues, indicating a general endothelial metabolic programme. Within bone, this endothelial state expands following high-fat diet and is suppressed during injury. Together, these findings redefine skeletal endothelial heterogeneity and establish the proposed type R population as part of a venous continuum.

## Full Text Availability

The license terms selected by the author(s) for this preprint version do not permit archiving in PMC. The full text is available from the preprint server.

